# Resistance gene identification from Larimichthys crocea with machine learning techniques

**DOI:** 10.1038/srep38367

**Published:** 2016-12-06

**Authors:** Yinyin Cai, Zhijun Liao, Ying Ju, Juan Liu, Yong Mao, Xiangrong Liu

**Affiliations:** 1 School of Information Science and Technology, Xiamen University, Xiamen, Fujian 361005, China; 2 State Key Laboratory of Large Yellow Croaker Breeding, Ningde Fufa Fisheries Company Limited, Ningde, 352000, China; 3 Department of Biochemistry and Molecular Biology, School of Basic Medical Sciences, Fujian Medical University, Fuzhou, 350122, China; 4 School of Aerospace Engineering, Xiamen University, Xiamen, Fujian 361005, China; 5 College of Ocean and Earth Sciences, Xiamen University, Xiamen, 361102, China

## Abstract

The research on resistance genes (R-gene) plays a vital role in bioinformatics as it has the capability of coping with adverse changes in the external environment, which can form the corresponding resistance protein by transcription and translation. It is meaningful to identify and predict R-gene of Larimichthys crocea (L.Crocea). It is friendly for breeding and the marine environment as well. Large amounts of L.Crocea’s immune mechanisms have been explored by biological methods. However, much about them is still unclear. In order to break the limited understanding of the L.Crocea’s immune mechanisms and to detect new R-gene and R-gene-like genes, this paper came up with a more useful combination prediction method, which is to extract and classify the feature of available genomic data by machine learning. The effectiveness of feature extraction and classification methods to identify potential novel R-gene was evaluated, and different statistical analyzes were utilized to explore the reliability of prediction method, which can help us further understand the immune mechanisms of L.Crocea against pathogens. In this paper, a webserver called LCRG-Pred is available at http://server.malab.cn/rg_lc/.

Larimichthys crocea is a primary economic fish species in China^1^, belonging to vertebrates. However, with the expansion of breeding scale, in particular the abuse of antibiotics, parasite as well as viruses and bacteria^1^,^2^,^3^, pathogens have become a major constraint in the sustainable development of aquaculture of L.Crocea. Resistance genes play a key role in L.Crocea’s immune system by transcribing to form resistance protein that contain Antimicrobial peptides (AP), Major histocompatibility complex (MHC), Immunoglobulin (Ig), Natural resistance associated macrophage protein (Nramp), Interferon (IFN), Lectin, Interleukins (ILs), tumour necrosis factors (TNFs), Lysozyme and etc. The expression of these genes can empower the organism against drugs or malnourished environment, such as antibiotics and communicable diseases, which are commonly used as selective genetic markers for developing excellent antibody strain. Despite advances in science, substantial genomic and transcriptome sequences call for genetic analyses in Larimichthys crocea^4^, and research on R-genes and R-gene-like genes can offer helpful understanding about the defense mechanisms of L.Crocea. These can not only meet breeding needs, but also the needs of life.

Certain methods have been utilized for R-gene mining, including experiment methods like protein/gene fusion^5^,^6^, sequence assembly^4^,^7^, sequence alignment/similarity^8^,^9^, and structure-based approach^10^,^11^, etc. Because of biological mining methods are time-consuming and expensive for genome identification, machine learning methods are developed much more efficiently in classification and prediction of R-gene. The classifiers^12^, e.g. Support vector machine^13^,^14^,^15^,^16^,^17^, Naive bayes^18^,^19^ and Random forest^20^,^21^,^22^ were applied. Despite recent advances and applications mainly focus on plant resistance genes such as Xia *et al*.^13^ and Torres-Avilés *et al*.^23^ predicted R-gene in rice and tomato separately, and NBSPred^24^ was proposed to predict R-gene of plant. Lii *et al*.^25^ and Thorsten *et al*.^26^ suggest that there exist several emerging similarities in plant R-gene and animal innate immune receptor complexes. Robertsen^27^ found that the IFNs producing cells of fishes and IFNs gene structure were similar to those in mammals, and the deduced protein of fishes was highly homologous to mammalian. This means that a limited number of all known R-gens can be a likely explanation for identifying the immune system of L.Crocea. Considering these and other similarities, as a solution, machine leaning was used to model all reviewed resistance genes in all species, and the model was evaluated and applied to identify L.Crocea for novel R-gene.

This paper aims to identify and analyze the resistance genes of Larimichthys crocea so as to improve its own immune system to fight against the invasion of pathogens. In view of the specific functional classes of proteins with common structure and physical-chemical characteristics, we extract feature information from all known R-gene sequences with machine learning methods, and classification algorithms are adopted for identification of the gene fragment separately. Potential rules of the sequences could be acquired by studying the reviewed sequences, and the same properties were able to confirm by using the classifier model we obtained to classify the unknown sequence. Moreover, different feature extraction methods and classification methods were compared, and the results and differences of the prediction are discussed and analyzed. In addition, the quality of the prediction was verified. The main flowchart of the process is given in Fig. 1. In short, experiments demonstrate that the proposed methods, especially the SVMProt-RF by using SVM-Prot^28^,^29^ combined with Random forest, could be utilized for the prediction of novel R-gene.

## Results

### Comparative Analysis

#### Sampling method Comparative Analysis

Firstly, on the basis of SVM-Prot feature method, we compared the performance of original samples (Ω_*0*ri*glR*−*g*_) and samples after two sampling strategies (Ω_*tr*_ and Ω_*wtr*_) separately under Random forest classifier, where all other parameters are the same. Table 1 shows the results based on three different sampling methods. As we can see, given that the number of non-R-gene is greater than R-gene, it makes no sense if R-gene was classified as non-R-gene, though it gets higher accuracy. Besides, weighted random sampling contributes to the best result, which is good for establishment of a better performance classifier.

#### Multi-Classifier Comparative Analysis

**Figure 2:**
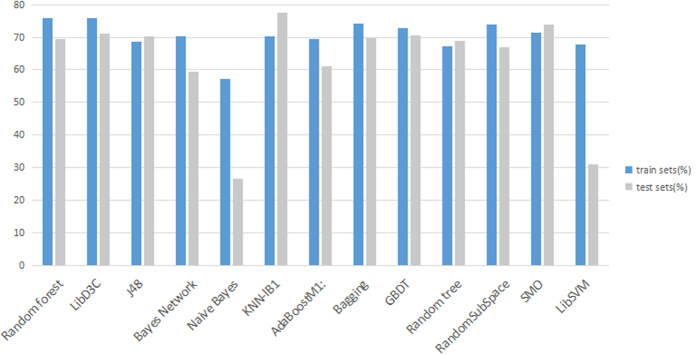
Performance of test sets on different classifiers.

In order to demonstrate the validity of the classification results of R-gene sequence in the Random forest algorithm, we compare the results of Ω_tr_ treated by SVM-Prot feature under different classifiers. To get the objective evaluation, we adopt both test set Ω_*test*_ and 10-fold cross-validation to verify the classification effect, as shown in Table 2 and Fig. 2. Visibly, the results of Random forest, LibD3C^30^, Bagging, Gradient Boosting Decision Tree (GBDT) and RandomSubSpace algorithm we obtained are better than others, their accuracies being 75.88%, 76.00%, 74.07%, 72.79% and 74.02% respectively, as shown in Table 2. In view of the performance of classifier, the sensitivity of J48, KNN-IB1, Random tree, GBDT and SMO are all less than 72%, that is, the model is less than 72% for classifying R-gene correctly, even if the total accuracy of some of these methods is very high. Besides, the sensitivities of Bayes Network, Naive Bayes, and LibSVM are higher than 80%, but their low specificities result in a serious false positive problem when identifying the R-gene. Different from the above classifiers, Random forest, LibD3C, AdaboostM1, bagging and RandomSubSpace with the guarantee of high sensitivity have an acceptable specificity. In addition, Random forest and LibD3C work better considering the Mcc, total accuracy and ROC Area. Furthermore, for the time consumed, LibD3C is 36 times more than Random forest with the same parameters. For the test set, KNN-IB1 achieved a higher accuracy rate of 77.5998% while Random forest 69.347%, as can be seen in Fig. 2, which can only indicate that KNN-IB1 has a higher classification accuracy of non-R-gene. Therefore, the function of Random forest classifier shows better with comprehensive consideration.

#### Multi-Feature Comparative Analysis

In this section, feature extraction methods are compared in our experiment on the basis of Random forest classifier, including the 188-D constructed from SVM-Port features, Pse-AAC^31^ features and 473-D features, as shown in Table 3. The strengths of the 188-D feature extraction algorithm is obvious, which obtains higher accuracy as well as higher sensitivity and specificity, better than the other two feature extraction algorithms. The second part of Table 3 denotes the accuracy of the training set and test set in 188-D features and Pse-AAC and 473-D feature method under the Random forest classifiers. And the accuracy of the test set of Pse-AAC reached 60.913% while SVM-Port features reached 69.347%, and 473-D features reached 55% respectively. We can learn that SVM-Prot features combined with Random forest have the best result among these algorithms through synthetical consideration. Here we call it SVMProt-RF method.

### Identification R-gene from Larimichthys crocea

**Figure 3:**
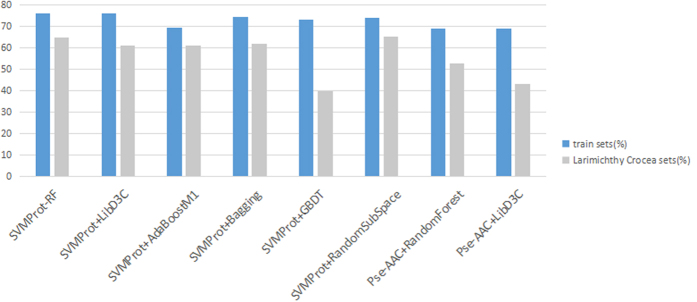
Prediction results of L.Crocea on different classification models.

To get a better understanding of Larimichthys crocea immune system for future breeding and disease prevention, an effective support and recognition of the resistance genes of L.Crocea is particularly crucial. In our experiments, a combined classification model was developed by identifying all reviewed R-gene, and it was applied to screen the R-gene of L.Crocea. As for the selection of the original data of prediction model, we used the protein sequence coded by R-gene based on the following conditions: R-gene expresses the resistance function through the protein product directly; protein sequence consists of 20 amino acid with abundant physicochemical properties, while nucleotide sequence consists of only 4 elements, which is not conducive to the feature extraction. Here, we obtained multiple hybrid prediction models with higher accuracy after a series of comparison as demonstrated before. Ω_*LC*_ (sequence of L.Crocea) was predicted based on these models. A comparison was made between SVMProt-RF method and others as well. Figure 3 gives the results of the prediction. As we can see, 64.64% R-gene existent in the sequences of L.Crocea while 61.01%, 61.12%, 61.68%, 39.74%, 65.16%, 52.70% and 43.20% were respectively obtained in others. Furthermore, Table 4 shows the prediction results of Ω_*LC*_ applied by Ω_*0*ri*glR*−*g*_ model, Ω_*tr*_ and Ω_*wtr*_ model, their prediction results taking up 45.30%, 64.64% and 54.39% respectively.

A comparative table of SVMProt-RF and NBSpred prediction is given in the Table 5, since there exist obvious similarities of pathogen-associated molecular patterns (PAMPs) in animals and plants, especially the plant receptors resembling mammalian Toll-like receptors (TLR) or cytoplasmic nucleotide-binding oligomerization domain leucine-rich repeat (LRR) proteins^26^, and NBSpred is a web server for predicting nucleotide binding site lucine-rich repeat proteins (NBS-LRR) of plant^24^. SVM method is used to extract features of datasets by calculating six compositional attributes, including amino acid frequency, dipeptide frequencies, tripeptide frequencies, multiplet frequencies and hydrophobicity composition^24^. Total, 9801 sequences are identified as R-gene and R-gene-like genes through SVMProt-RF. NBSPred only detected 2.544% sequences as R-gene from L.Crocea dataset. Distinct differences remain in plants and vertebrates, such as plants do not own specific immunity and cannot produce immunizations because they lack circulatory blood system like an animal. So, we can find that one prediction model can identify R-gene of plants accurately but fails to predict R-gene of L.Crocea.

## Discussion

In this paper, after comparison among different feature extraction methods and classification algorithms, the SVM-Prot feature extract method and random forests classification algorithm were combined (SVMProt-RF) to preliminarily mine the resistance gene of the whole protein data, which proves to achieve the best results. And further screening was conducted on the acquired resistance gene to determine the relationship between the candidate sequence and the resistance trait. The work was divided into the following parts: the establishment of resistance data sets, the feature extraction, the sampling of imbalanced data sets and the comparison of resistance genes classification models. In comparison with other previously mentioned works and methods, we can reach the conclusion that our methods have the following advantages:

(1) It reduce the redundancy of R-gene samples, and optimize efficiency by keeping the original data information.

(2) Feature extraction based on datasets that contains resistance genes of all reviewed species and the prediction of R-gene of L.Crocea are more accurate.

(3) Compared with other classifiers, the result of SVMProt-RF method associated with weight random-sampling shows that the model has a better sensitivity and specificity, and better adaptability to identify R-gene.

(4) It Can be used to predict the resistance genes of more candidate sequences, and verify the correlation between them with biological experiment.

The establishment of the model is of great significance for the subsequent resistance gene discovery and its evolution, regulation and pathway analysis. What’s more, for the immune system-related genes of Larimichthys crocea, further exploration is still required.

## Method

### Data preprocessing

The original R-gene sequences were retrieved from Uniprot database^32^, which has been reviewed by experimentation. The dataset is composed of 13,959 sequences that contains all species like zoon, plants and fungi, denoted as Ω_*0*ri*glR*−*g*_. Each R-gene class, nevertheless, contains a lot of duplicate sequences that cause excessive redundancy. Therefore, CD-HIT was utilized to remove redundancy in positive dataset, which has been used in the realm of bioinformatics^33^,^34^. Considering the following algorithm: First, sort out all sequences according to their length; then form the classes by sequentially processing the length sequence. If the similarity of new sequence was higher than the existing class in threshold, the new sequence was added to this class, otherwise make it as a new class. Finally, 6720 R-gene were obtained with similarity below 70% after CD-HIT, denoted as Ω_*R*−*g*_:


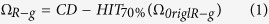


The negative sample was acquired from PFAM families due to the intimate relationship between R-gene and its protein sequence. No-duplicates PFAM of R-gene were removed from the whole PFAM families database. We got negative families here, and the longest sequence of proteins was fetched in each negative families. 10028 non-R-gene sequences were involved, denoted as Ω_*NR*−*g*_. Thus the training dataset Ω is denoted as follows:


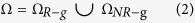


where Ω contains a total of 16,748 sequences. The prediction datasets of Larimichthys crocea that consist of 18,018 sequences are collected from Uniprot database^32^ as well. To describe it simply, we denoted it as Ω_*LC*_.

### Feature extraction algorithm

#### SVM-Prot features

SVM-Prot is a web server for protein classification. It constructs 188-D features for protein sequences description and classification^28^,^29^. The features have been applied successfully in several protein identification works, such as cytokines^35^,^36^ and enzymes^37^,^38^. The extracted features include hydrophobicity, normalized van der Waals volume, polarity, polarizability, charge, surface tension, secondary structure and solvent accessibility^28^. For each of these 8 types of physical-chemical properties, some feature groups were designed to describe global information of protein sequences. These feature groups contain composition (C), transition (T) and distribution (D)^14^,^28^. C expresses a percentage of the amino acids of particular property over total amino acid sequence. T is the frequency of amino acids of particular property that are intimately next to another amino acid of particular property. D depicts the position of amino acids of particular property in their sequences. Thus, the dimension of each feature vector is 21 (denoted as *D*_*eachV*_). In addition, considering amino acid composition (denoted as *H*_acc_), the protein structure is composed of 20 amino acids: A, C, D, E, F, G, H, I, K, L, M, N, P, Q, R, S, T, V, W, Y^39^,^40^. So the dimension of 188-D features is


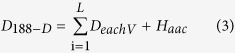


where *L* is the number of features. The features of Ω and Ω_*LC*_ were extracted. Table 6 shows a part of the results of PSBA1 R-gene in Acaryochloris marina.

#### Pseudo amino acid composition features

Pseudo amino acid composition features (Pse-AAC)^41^ as an efficient computation tool has been diffusely leveraged for protein sequences in predicting protein structures and functions^31^,^41^, as well as DNA and RNA sequences^42^. To describe it distinctly, we assume a R-gene sequence *R*, expressed as:


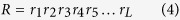


here, *L* denotes the length of the sequence and *r*_*i*_ (*i* = 1, 2, …, *L* is the position of residue in *R*. Besides, given the different amphiphilic features of proteins, the Pse-AAC feature of *R* can be defined as the following vector^41^,^42^:


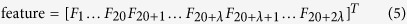



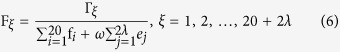



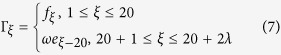


where *f*_*i*_ (1 ≤ *i* ≤ 20) denotes the frequency of the 20 amino acids in *R*, and *λ* is the top counted rank of the correlational protein sequences. We have a 30 dimension feature vector in this experiment. *ω* represents the weight factor, and *e*_*j*_ depicts the correlation factor among residues of protein sequences. Features of R-gene were extracted by this feature representation method, which sufficiently incorporates the effects of sequence order.

### Data Balancing

The unbalanced data problem always has huge impact on the result of the classification^43^. The classifiers tend to have a higher recognition rate for the majority class, which make it hard to identify the minority class correctly^44^,^45^. What we want is to eliminate the over fitting problem caused by unbalanced data. The commonly used method is sampling^46^, including under-sampling and over-sampling.

Since it is easy to obtain reviewed R-gene but not the non-R-gene, which incurs serious class imbalance problem and affects the performance of the classifier, two sampling methods are used in this paper to find out the best performance. One is random-under-sampling. The balance of the train sets is realized by random sampling of large class set, where the number of large class sets equals the small class sets. Here we get 6720 sequences each for Ω_*NR*−*g*_ and Ω_*R*−*g*_ as train sets, denoted as Ω_tr_, and 3308 negative sequences remain as test sets Ω_test_. Another method we applied is weighted random sampling^47^, balancing the dataset by adding different weights to the unbalanced samples. Seeing that the ratio about Ω_*R*−*g*_ and Ω_*NR*−*g*_ is approximately equal to 7:10, weight factor 10 and 7 were added to the Ω_*R*−*g*_ and Ω_*NR*−*g*_ separately, so 16748 train sets were obtained, denoted as Ω_wtr_.

### Classifier selection and tools

#### Random forest

Random forest is a kind of classifier which is trained and predicted by a number of trees, as proposed by Leo Breiman^48^. Numerous advantages have been listed than other algorithms, including noise-ability, avoiding over-fitting, being able to handle high dimensional (feature) data and etc. The essence in this algorithm is an improvement based on the decision tree. An object can be categorized into a class, when the class follows the principle of the judgment based on every decision tree in the forest. The classification ability of the single tree would be marginal, but the probability of being classified properly is greatly enhanced after random generation of a large number of decision trees. In this study, R-gene is a binary classification, so all decision trees are binary tree.

#### WEKA

WEKA is one of the well-known data mining platform (http://www.cs.waikato.ac.nz/ml/weka/) that are utilized for data analysis and model prediction. Several machine learning algorithms were gathered as tools. Cross-validation is provided by WEKA. In this study, we utilize its classification function to establish a model of Ω_tr_, and its test sets Ω_test_ to verify the precision of the model. Thirteen classifiers are selected for this paper.

### Measurement

Sensitivity (SN), specificity (SP), overall accuracy (Acc) and Matthew’s correlation coefficient (Mcc) are usually applied in bioinformatics^49^,^50^,^51^,^52^,^53^,^54^,^55^ to measure the function of the classifier. Given datasets 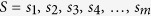, *m* is the number of samples. Based on the confusion matrix of binary classification performance of R-gene (shown in Table 7), we have:


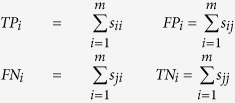


where *TP*_*i*_, *FP*_*i*_, *TN*_*i*_, *FN*_*i*_ denote the numbers of true positive instances, false positive instances, true negative instances and false negative instances respectively. The first subscript of *s*_*ii*_ indicates the prediction result and the second indicates the true class of sample *s*_*m*_. And we have^14^,^56^:


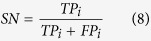



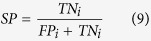



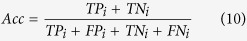



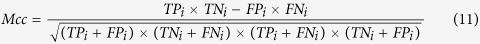


## Additional Information

**How to cite this article**: Cai, Y. *et al*. Resistance gene identification from Larimichthys crocea with machine learning techniques. *Sci. Rep.* **6**, 38367; doi: 10.1038/srep38367 (2016).

**Publisher's note:** Springer Nature remains neutral with regard to jurisdictional claims in published maps and institutional affiliations.

## Figures and Tables

**Figure 1:**
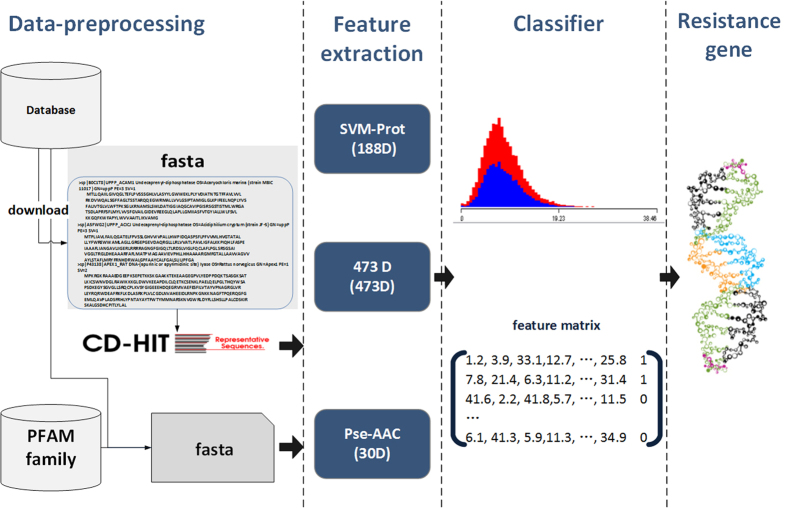
The main flowchart of the identification process.

**Table 1:** Results based on three different sampling methods using random forest.

Sampling Method	Training set		Accuracy			
	Resistance gene	Non-Resistance gene	SN	SP	Accuracy (%)	ROC Area
Original instance	6720	10028	0.821	0.696	77.0898	0.855
Random-under-sampling	6720	6720	0.831	0.687	75.878	0.850
Weighted random-sampling	6720	10028	0.767	0.761	76.3974	0.854

**Table 2:** Performance comparison of different classifier.

Classifier	Attributes	SN	SP	Mcc	Accuracy (%)	ROC Area
Random forest	13440	0.831	0.687	0.523	75.878	0.850
LibD3C	13440	0.820	0.700	0.524	76.0045	0.846
J48	13440	0.688	0.683	0.371	68.5491	0.678
Bayes Network	13440	0.810	0.597	0.417	70.3646	0.761
Naive Bayes	13440	0.882	0.264	0.185	57.2768	0.690
KNN-IB1	13440	0.639	0.765	0.408	70.2158	0.706
AdaBoostM1	13440	0.782	0.605	0.393	69.3601	0.763
Bagging	13440	0.786	0.696	0.483	74.0699	0.822
GBDT	13440	0.718	0.705	0.456	72.7902	0.818
Random tree	13440	0.673	0.672	0.346	67.2842	0.673
RandomSubSpace	13440	0.819	0.662	0.486	74.0179	0.826
SMO	13440	0.677	0.749	0.427	71.2798	0.713
LibSVM	13440	0.947	0.307	0.331	62.7232	0.627

**Table 3:** Performance comparison of 188-D features and 473-D features.

Feature extraction method	Dimension	Training set		Accuracy			
		Resistance gene	Non-Resistance gene	SN	SP	Mcc	Accuracy (%)
188-D	188	6720	6720	0.831	0.687	0.523	75.878
Pse-AAC	30	6720	6720	0.761	0.627	0.392	69.4345
473-D	473	178	226	0.371	0.752	0.133	58.4158
**Feature extraction method**	**Dimension**	**test set**					
		**Resistance gene**		**Non-Resistance gene**		**Accuracy (%)**	
188-D	188	0		3308		69.347	
Pse-AAC	30	0		3308		60.9129	
473-D	473	20		20		55.0	

**Table 4:** Prediction results of Ω_LC_ under different data balancing models.

Prediction model	Accuracy		
	TP Rate	TN Rate	Accuracy (%)
Ω_0riglR−g_ model	0.453	0.547	45.3047
Ω_tr_ model	0.646	0.354	64.6409
Ω_wtr_ model	0.546	0.454	54.3956

**Table 5:** Comparison of SVMProt-RM and NBSPred prediction for R-gene of L.Crocea.

Dataset	Number of sequences	SVMProt-RF prediction	NBSPred prediction
L.Crocea Dataset	18018	9801	457 17964 (total number after NBSPred)
Accuracy (%)		54.3956	2.5440

**Table 6:** Feature of PSBA1 R-gene in Acaryochloris marina.

Property	Value of feature vector						
amino acid composition	9.3664	0.2755	1.6529	3.5813	6.0606	8.5399	3.8567
	7.1625	0.8264	12.1212	4.6832	3.5813	4.9587	2.7548
	3.3058	9.3664	6.8871	5.5096	2.7548	2.7548	
Hydrophobic	15.7025	45.7300	38.5675	12.9834	12.4309	37.5690	1.6529
	29.2011	62.8099	82.6446	97.5207	0.5510	24.7934	49.5868
	73.0027	100.0	1.6529	25.3443	52.066	75.7576	99.1735
Van der Waals volume	0.2755	28.9256	50.9642	74.1047	99.4490	41.3223	39.1185
	19.5592	33.9779	17.6796	12.7072	0.2755	23.4160	45.1791
	72.1763	99.4490	0.5510	23.1405	48.4848	73.8292	100.0

**Table 7:** Confusion matrix of binary classification performance of R-gene.

Classification	Positive instance of prediction	Negative instance of prediction
Positive instance	*TP*_*i*_	*FN*_*i*_
Negative instance	*FP*_*i*_	*TN*_*i*_
